# A Retrospective Analysis of National Kidney Registry Integration at a Single Center in the Northern Great Plains

**DOI:** 10.1155/joot/6424483

**Published:** 2025-11-11

**Authors:** Els Reuvekamp, Benjamin Limburg, Kaleb Dobbs, Sujit Vijay Sakpal

**Affiliations:** ^1^University of South Dakota Sanford School of Medicine, Sioux Falls, South Dakota, USA; ^2^Avera Medical Group Transplant & Liver Surgery, Avera McKennan Hospital & University Health Center, Sioux Falls, South Dakota, USA; ^3^Department of Surgery, University of South Dakota Sanford School of Medicine, Sioux Falls, South Dakota, USA; ^4^Department of Internal Medicine, University of South Dakota Sanford School of Medicine, Sioux Falls, South Dakota, USA

## Abstract

**Background:**

Despite the National Kidney Registry's (NKR) widespread adoption, limited data exist to explain center-specific trends in live kidney donation (LKD) in rural areas.

**Methods:**

A retrospective review of 1776 referrals (894 before and 882 after NKR integration on February 1, 2018) for LKD at our center between June 1, 2012, and May 31, 2022, was performed. LKD referrals were comparatively analyzed between pre- and post-NKR phases and followed through subsequent evaluation, donation, or termination of donor candidacy.

**Results:**

Both pre- and post-NKR, donors were most likely to be White (93.2% vs. 89.4%, *p*=0.33), women (73.0% vs. 66.0%, *p*=0.51), from South Dakota or neighboring states (97.3% vs. 89.4%, *p*=0.11), and employed (95.9% vs. 95.7%, *p*=0.99). Following NKR affiliation, our center experienced a significant increase in LKDs (74 vs. 47, *p*=0.008), most notably from nondirected (9 vs. 1, *p*=0.04) and rural (37 vs. 18, *p*=0.099) donors and those from socioeconomically disadvantaged (Area Deprivation Index: 3 vs. 4 state decile, *p*=0.055, and 47 vs. 54 national percentile, *p*=0.056) communities. Post-NKR donor pool showed greater diversity in educational backgrounds and lower rates of tobacco and illicit drug use. Also, post-NKR referrals were evaluated, on average, 10 days sooner (66 vs. 76 days and *p*=0.01) and were less likely to retract or be lost to follow-up after evaluation (25.1% vs. 32.7% and *p*=0.04).

**Conclusion:**

NKR potentiates expanded LKD at rural transplant centers. Efforts to increase LKD among men and People of Color remain areas of opportunity.

## 1. Introduction

Live donor kidney transplantation (LDKT) offers renewed hope to patients with end-stage renal disease (ESRD) by providing shortened wait times to transplantation and improved outcomes compared to deceased-donor KT (DDKT), yet current rates of live kidney donation (LKD) do not meet the demand for donor organs [1]. Organ shortage significantly hampers the ultimate ESRD treatment, with racial, sociodemographic, and geographic factors contributing to lower transplant rates [2]. Existing literature often focuses on financial factors and altruism affecting LKD, but sociodemographic influences on potential donors are less studied. Efforts by governments and organizations to boost LKD rates generally address financial and health-related barriers without considering gender, culture, or racial factors [3]. Despite well-intentioned policies like tax credits and paid leave for donors, these have had minimal impact on LKD rates in the United States [4]. This suggests that unexplored barriers may be undermining the effectiveness of programs designed to improve LKD access.

With over half of South Dakotans living in nonmetro areas [5], the state's rurality would be expected to impact access to LKD and LDKT. Most recently, the 2021 KAS250 organ allocation changes have resulted in decreased rates of DDKT in nonmetropolitan recipients [6]. While rurality may not precipitate longer wait times for KT in predominantly White study populations [7], it has been found that race notably impacts this relationship, as racial minorities residing in rural communities are significantly less likely to undergo transplantation than their White rural counterparts [8]. Most existing transplant-specific research into race and rurality compares Black and White populations, underscoring a need to further investigate the experiences of non-Black minority patients, such as Indigenous Peoples (IP) [9].

The relevance of these existing findings may be further limited by specific barriers to organ donation and transplantation that are present in South Dakota (SD). For instance, transportation barriers delaying medical care are more likely to affect racial and ethnic minority groups, along with individuals living in rural areas [10], a finding expected to have an outsized impact on the IP of SD [5].

Sociodemographic factors intersect with logistical barriers to organ donation. While some aspiring donors may be able to donate a kidney directly to an intended recipient, many who wish to do so are not a match or have no access to a compatible recipient. The National Kidney Registry (NKR) aims to increase LKDs by improving access to paired-exchange and nondirected donation (NDD). This study investigates the barriers to LKD by examining how NKR affiliation impacted the process of LKD at the Avera Transplant Institute (ATI), a Sioux Falls, SD-based multiorgan transplant center, serving as the largest solid-organ transplant center within a nearly 200-mile, mainly rural sociodemographic radius. Through analysis of characteristics of potential and actual donors before and after NKR affiliation at ATI, this study intended to determine the impact of NKR affiliation on improving access to LKD.

## 2. Methods

### 2.1. Data

Retrospective review and analysis of all individuals who self-referred to participate in LKD at ATI over a 10-year period (June 1, 2012, to May 31, 2022) was performed. Patient health information was retrieved from ATI's OTTR Organ software system. Data were categorized on the basis of each patient's date of referral, with February 1, 2018, marking the date of ATI's affiliation with NKR. The pre-NKR period consisted of 2071 days (5 years and 8 months), and the post-NKR period consisted of 1581 days (4 years and 4 months).

### 2.2. Donor Screening Process

**Figure 1:**
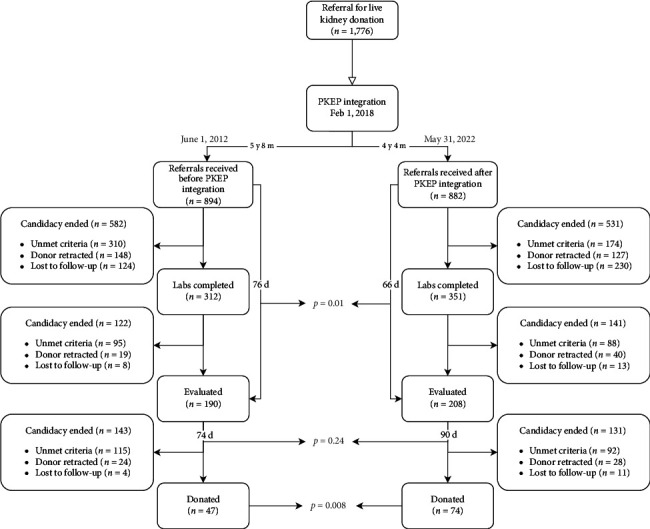
Flowchart of self-referred individuals progressing through the donor workup process to kidney donation before and after National Kidney Registry (NKR) integration within Avera's live donor program.

Pre-NKR, most referrals completed intake via phone conversation with ATI staff, whereas referral intake was largely completed via online form post-NKR. The steps of the donor workup process that followed intake completion including laboratory testing, in-person evaluation, and selection committee approval or rejection were performed similarly for both cohorts (Figure 1).

The referral phase began with the potential donor's first contact with ATI. Donor intake served as an initial screening tool to determine baseline eligibility to donate through review by live donor program (LDP) coordinators based on inclusionary, exclusionary, and relative exclusionary criteria in consultation with transplant physicians as needed. Referred patients who passed the initial screening were informed of and consented to the workup process, starting with laboratory testing, by an LDP coordinator. Laboratory results were reviewed by a transplant physician, and patients whose results met criteria were invited to complete an in-person evaluation with the multidisciplinary transplant team. The evaluation process consisted of a psychosocial exploration by a licensed social worker and/or psychologist, a medical and surgical assessment by clinicians including a transplant nephrologist, surgeon, pharmacist, dietitian, and financial coordinator, with support from an independent live donor advocate. Upon evaluation, the multidisciplinary transplant committee conducted holistic reviews of the above information for each candidate, with selection and approval of donors constituting the final step of the donor workup process.

### 2.3. Donor Selection

Donor approval was dependent upon the successful stepwise progression of potential donors through the referral, evaluation, and donation stages of the LKD screening process. Subjects who were ultimately deemed ineligible to donate were considered “rejected,” with the reason for rejection being categorized as “criteria unmet,” “lost to follow-up (LTFU),” or “donor retracted.”

Participation in the LKD process was contingent upon each candidate's continued volunteerism and reciprocal communication with the LDP. Potential donors reserved the right to withdraw their candidacy at any time for any reason and could disclose a reason for withdrawal at their own discretion. To ensure protection of potential donors' right to withdraw, a persistent lack of reciprocal contact between a potential donor and the LDP resulted in termination of that potential donor's candidacy for LKD by the selection committee due to a LTFU. In cases where the potential donor could only be contacted sparsely, their candidacy was rejected based on failure to meet the inclusionary criterion of volunteerism. A final communication letter was sent to each candidate to notify them of this decision, after which the center would no longer reach out to the candidate so as to ensure strict adherence to ethical guidelines and maintain the individual's freedom from sentiments of pressure or coercion.

### 2.4. Rurality, Distance, and Area Deprivation Index (ADI)

Donors whose city of residence had a population of < 30,000 per the 2020 US Census were assigned as “rural,” for the purposes of this study. The distance from donors' residence zip codes to ATI's zip code was used to estimate donors' distances from our center. The Neighborhood Atlas ADI, which ranks Census blocks by socioeconomic disadvantage on the basis of income, education, employment, and housing quality, was used as a measure of donor's socioeconomic disadvantage [11]. Donors' geographical addresses were used to assign ADI numbers per the 2024 ADI data.

### 2.5. Analysis

Potential donor characteristics and LKD progression were compared using chi-square, *t*-test, and Kruskal–Wallis analysis for both the pre-NKR and post-NKR cohorts. Each potential donor's sociodemographic characteristics, medical status, and outcomes were recorded, and the prevalence of features was analyzed between cohorts. Avera Health's Institutional Review Board (IRB) approved this study [IRB protocol #: 2018.039-100559].

## 3. Results

A total of 1776 individuals referred themselves for LKD to ATI from June 1, 2012, to May 31, 2022, with 894 referrals received prior to NKR integration (February 1, 2018) and 882 referrals after (Figure 1). Following NKR integration, ATI saw more donations altogether (74 vs. 47, *p*=0.008) and increased retention of referred individuals at each stage of the donor workup process, with greater proportions of referrals undergoing evaluation (23.6% [208/882] vs. 21.3% [190/894], *p*=0.26) and evaluations donating (35.6% [74/208] vs. 24.7% [47/190], *p*=0.022). Compared to pre-NKR, postevaluation donor retraction or rejection decreased post-NKR (25.1% vs. 32.7%, *p*=0.04), augmenting donor retention. Post-NKR, referrals were more likely to be excluded due to LTFU prior to evaluation (44% vs. 21%, *p*=0.01). Patients underwent evaluation, on average 10 days sooner in the post-NKR period (66 vs. 76 days, *p*=0.01), yet experienced an average of 16 days longer waiting time between evaluation and donation (90 vs. 74 days, *p*=0.24). Both referrals and donations occurred more frequently in the post-NKR period, with one referral occurring approximately every 1.79 days post-NKR, compared to every 2.32 days pre-NKR (*p* < 0.001), and one donation occurring approximately every 21 days post-NKR, compared to every 44 days pre-NKR (*p* < 0.001). This yielded an increased average yearly referral rate (158 referrals/year vs. 204 referrals/year, *p*=0.63) and donation rate (8.3 donors/year vs. 17 donors/year, *p*=0.35).

Post-NKR, women accounted for a greater proportion of referrals (70.3% vs. 60.2%, *p*=0.02) and donations (73.0% vs. 66.0%, *p*=0.51) (Table 1). Despite these changes, NKR integration resulted in a net increase of 4 male donors (*p*=0.42). These changing donor demographics were accompanied by increases in the percentages of donors who were single (31.1% vs. 25.5%, *p*=0.54), living alone (25.7% vs. 14.9%, *p*=0.18), and without dependents (24.3% vs. 14.9%, *p*=0.25). Proportions of White patients increased among referrals (88.2% vs. 68.7%, *p*=0.0001) and donations (93.2% vs. 89.4%, *p*=0.33), and White referral-to-donation conversion increased from 6.8% to 8.9% (*p*=0.20) (Table 1). Referrals from IP decreased (67 vs. 121, *p*=0.04), yet there were more Indigenous donors (5 vs. 2, *p*=0.72). Indigenous referral-to-donation conversion increased from 1.7% to 7.5% (2/121 vs. 5/67, *p*=0.10) but remained lower than overall post-NKR referral-to-donation conversion (5/67 vs. 74/882, *p*=0.99).

**Figure 2:**
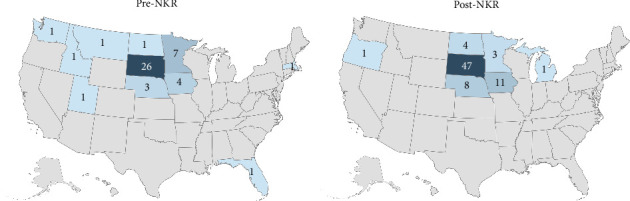
Comparison of donors' states of residence before and after Avera's affiliation with the National Kidney Registry (NKR).

In both periods, individuals from SD or neighboring states comprised the majority of referrals (794/894 vs. 766/882, *p*=0.22) (Table 1) and represented a greater percentage of donations following NKR integration (97.3% [72/74] vs. 89.4% [42/47] vs., *p*=0.11) (Figure 2). Despite this, there were decreased post-NKR referrals (40/882 vs. 81/894, *p*=0.0002) and donors (3/74 vs. 7/47, *p*=0.0455) from Minnesota (MN) seemingly due to the proximity of alternative NKR-affiliated transplant centers within the state. Post-NKR, donors were more likely to be from rural communities (50% [37/74] vs. 38.3% [18/47], *p*=0.099) (Table 1), and the average donor lived farther away from ATI (109 vs. 99 miles). Also, donors had a higher median state ADI decile (3 vs. 4, *p*=0.055) and national ADI percentile (47 vs. 54, *p*=0.056) post-NKR.

Among referrals, the most common relationship to the intended recipient in both periods was a relative (44.7% vs. 33.7%, *p*=0.0001) (Table 1). The greatest shift in relationship to intended recipients was seen in NDD, with an increase in referrals (13.5% vs. 2.8%, *p*=0.01) and donations (12.2% vs. 2.1%, *p*=0.04) post-NKR.

Donors of all educational backgrounds increased after NKR integration, with the most notable increase in donors whose highest level of education was a high school diploma/general educational development (GED) (21.6% vs. 10.6%, *p*=0.14) (Table 1) and those who had ≥ Bachelor degree (47.3% vs. 44.7%, *p*=0.85). Despite educational differences, no statistically significant difference was noted in employment statuses of post-NKR donors compared to pre-NKR, with employed individuals making up most donors in both periods (95.9% vs. 95.7%, *p*=0.99).

The post-NKR period saw increased referrals reporting daily alcohol use (14.1% vs. 3.7%, *p*=0.0001) and/or active illicit drug use (6.7% vs. 2.7%, *p*=0.0001) (Table 1). These changes were accompanied by higher rates of daily alcohol consumption among donors (14.9% vs. 4.3%, *p*=0.08), but lower rates of tobacco (6.8% vs. 8.5%, *p*=0.73) and illicit drug (4.1% vs. 6.4%, *p*=0.68) use. Unknown or unlisted lifestyle habits were most common during the pre-NKR period and among referrals, with rates of unknown/unlisted answers decreasing at each stage of the workup process.

## 4. Discussion

In just over four-and-a-half years following ATI's NKR affiliation, nearly twice as many LKDs were facilitated compared to the prior five years. With an increased likelihood of self-referred individuals becoming donors post-NKR, our study's findings support others' demonstrating higher rates of LKD at NKR-affiliated hospitals compared to those without NKR integration, evidently due to NKR's paired-exchange methodologies [12]. Donor retraction and LTFU, the most common reasons for candidate withdrawal in our study, highlight the importance of candidates' motivation, or lack thereof, to donate. Nevertheless, donor retention improved at each step of the LKD process, indicating that NKR affiliation enabled motivated candidates to donate past the incompatibility barrier with their intended recipient. In addition, NKR encouraged significant NDDs. NDD amplifies the benefits of direct-paired donation altogether, as the nondirected donors used to initiate transplant chains in the NKR's model result in ∼4.8 transplants for each NDD performed [13]. Furthermore, logistical planning of paired-exchange donation involving multiple centers explains post-NKR extended median duration after evaluation to donation.

Higher post-NKR donation rates at our center were accompanied by shifts in the demographic makeup of LKD candidates, with an increase in women donors exceeding the national average of 60% [14]. Hypothetically, gender differences in donation may be attributed to the financial implications of traditional gender roles, in which men must hold employment to support the household and are therefore less capable to donate than their female counterparts [15]. However, this contradicts the high rates of both women donors and employed donors identified in our study, as a majority of SD households claim women as breadwinners or co-breadwinners [16]. If partnership is used as a proxy for financial support, an increase in donors who were single and living alone post-NKR within our study may suggest that financial factors were improbable to have impacted candidates' ability to donate. Because relationship status and cohabitation also correspond to social support, our study's findings imply that donors' access to social support too was unlikely to impact their ability to donate. Combined, these findings suggest that neither financial nor social support theories explain the gender discrepancy in donation. Perhaps women's higher propensity to donate could be contextualized by factors such as gender-based expectations of women in patriarchal societies [15] and their greater emotional intelligence than men [17].

The racial makeup of our study's referral groups largely reflects the population demographics of SD, wherein Whites make up a majority (> 80%) of the state's population followed by IP (11.1%), Blacks (3%), and Asians (2.1%) [5]. Limited evidence currently exists explaining the unique needs of Indigenous organ donors, though it's been claimed that Indigenous transplant recipients prefer an LDKT over a DDKT [18]. Also, the same study found that IP in need of a KT were not as likely to have a family member willing to donate compared to the general patient population. Cultural characteristics could contribute to the low Indigenous referrals seen in our study. Infrastructural barriers potentially impede organ donation among IP, as the majority of IP in SD reside across its nine Indian Reservations which suffer from unemployment and poverty [19]. Due to a scarcity of Blacks within SD, our study could not reliably confirm findings of previous studies which found insignificance of NKR affiliation on donation from Black live donors [20].

Changes in the geographic makeup of this study's donor pool following NKR affiliation suggest its effectiveness to serve the needs of patients in geographically isolated, rural communities. An increase in donors from SD, Nebraska, Iowa, and North Dakota alongside a decrease in donors from MN—a state which has multiple, NKR-affiliated transplant programs—indicates that new donors had possibly underserved options to donate (Figure 2) and that NKR's paired-exchange model provides more possibilities than the traditional reciprocal match process, thereby enhancing access to donation [21]. ADI has been established as a valid metric of socioeconomic deprivation [11], and higher ADIs among donors in our study reflect improved access to transplantation following NKR integration. This, combined with the aforementioned changes in patient rurality and social and financial support, suggests the effectual penetrance of NKR in allaying sociodemographic barriers to donation [22].

While our findings mirror others, which found that potential donors who have college degrees are more likely to donate than those who do not [20], the differences in educational level of actual donors post-NKR were less pronounced. In fact, following NKR integration, we saw increased donation rates across all and diverse educational levels which was a meaningful deviation from previous claims that NKR-affiliated hospitals tend to have more donors with higher education [12]. These findings may question the directionality of the relationship between NKR affiliation and donors' educational backgrounds, as we saw the greatest increase in donation among candidates who had a high school diploma/GED, perhaps due to our unique sociodemographic landscape.

NKR integration led to the adoption of an online intake questionnaire inclusive of lifestyle-related inquiries and precision in donor assessment [12]. At our center, it enabled early identification of tobacco and illicit drug use among a large number of self-referring individuals, thereby efficaciously channeling our resources to accommodate the increase in the numbers of overall referrals and donations.

Rurality in the Northern Great Plains embracive a large proportion of IP population across various Indian Reservations constituted the 10-year cohort of our distinctive review. This said, a single-center, retrospective study inclusive of a period within the COVID-19 pandemic had its limitations. Also, less comprehensive intake protocols prior to NKR integration led to a higher incidence of unknown or unlisted potential donor characteristics. Notably, while LTFU was a common reason for termination of donor candidacy—and became even more common in the post-NKR period—reasons for LTFU and self-withdrawal from the LKD process were difficult to ascertain. Data gathered about withdrawal were limited by the ethical responsibility to ensure an LKD process that upholds each individual's right to withdraw and that is free from any acts which may be perceived as placing undue pressure upon its participants. Candidates were not asked to provide a reason for withdrawal, and only the reasons for withdrawal which were freely volunteered by the candidate were gathered, resulting in a few known reasons for withdrawal or LTFU.

LKD remains a vital avenue toward combating the donor organ shortage. Our study aimed to explore trends in the sociodemographic makeup of potential and actual live kidney donors at a rural transplant center before and after NKR affiliation and found that NKR enabled expansion of LKD across the regional socioeconomic strata with a potential to overcome innate barriers of rurality. Future research involving a combination of IP and rurality will effectively assist to increase LKD among men and People of Color.

## Figures and Tables

**Table 1:** Demographic comparisons of potential and actual donors before and after National Kidney Registry affiliation at Avera Transplant Institute's live donor program.

	Referrals		Evaluations		Donations	
	Pre-NKR *n* (%)	Post-NKR *n* (%)	Pre-NKR *n* (%)	Post-NKR *n* (%)	Pre-NKR *n* (%)	Post-NKR *n* (%)
All	894	882	190	208	47	74
Age ± SD (years)	44 ± 13.6	41 ± 13.3	45 ± 13.1	43 ± 13	42 ± 13	44 ± 12.1
BMI ± SD (kg/m^2^)	28.4 ± 5.9	27.6 ± 4.3	27.1 ± 4.4	26.9 ± 3.7	26 ± 3.5	27.2 ± 3.3
Median state ADI decile	—	—	—	—	3	4
National ADI percentile	—	—	—	—	47	54
Woman	538 (60.2)	620 (70.3)	128 (67.4)	147 (70.7)	31 (66.0)	54 (73.0)
Man	350 (39.1)	261 (29.6)	62 (32.6)	61 (29.3)	16 (34.0)	20 (27.0)
Not listed	6 (0.7)	1 (0.1)	—	—	—	—
Employed	535 (59.8)	749 (84.9)	166 (87.4)	186 (89.4)	45 (95.7)	71 (95.9)
Unemployed	109 (12.2)	117 (13.3)	22 (11.6)	19 (9.1)	2 (4.3)	3 (4.1)
Not listed	250 (28.0)	16 (1.8)	2 (1.1)	3 (1.4)	—	—
In a partnership	449 (50.2)	531 (60.2)	131 (68.9)	144 (69.2)	35 (74.5)	51 (68.9)
Single	245 (27.4)	346 (39.2)	57 (30.0)	63 (30.3)	12 (25.5)	23 (31.1)
Not listed	200 (22.4)	5 (0.6)	2 (1.1)	1 (0.5)	—	—
Cohabitant	470 (52.6)	567 (64.3)	153 (80.5)	151 (72.6)	40 (85.1)	53 (71.6)
Living alone	74 (8.3)	283 (32.1)	19 (10.0)	53 (25.5)	7 (14.9)	19 (25.7)
Not listed	350 (39.1)	32 (3.6)	18 (9.5)	4 (1.9)	—	2 (2.7)
With dependents	486 (54.4)	620 (70.3)	145 (76.3)	154 (74.0)	40 (85.1)	55 (74.3)
Without dependents	150 (16.8)	247 (28.0)	42 (22.1)	51 (24.5)	7 (14.9)	18 (24.3)
Not listed	258 (28.9)	15 (1.7)	3 (1.6)	3 (1.4)	—	1 (1.4)
White	614 (68.7)	778 (88.2)	161 (84.7)	184 (88.5)	42 (89.4)	69 (93.2)
Black	6 (0.7)	5 (0.6)	—	—	—	—
Asian	5 (0.6)	5 (0.6)	1 (0.5)	1 (0.5)	—	—
Indigenous persons	121 (13.5)	67 (7.6)	13 (6.8)	20 (9.6)	2 (4.3)	5 (6.8)
Native Hawaiian	—	1 (0.1)	—	—	—	—
Multiracial	—	5 (0.6)	—	—	—	—
Hispanic	12 (1.3)	21 (2.4)	6 (3.2)	3 (1.4)	2 (4.3)	—
Not listed	136 (15.2)	—	9 (4.7)	—	1 (2.1)	—
Primarily English speaking	367 (41.1)	859 (97.4)	97 (51.1)	204 (98.1)	45 (95.7)	72 (97.3)
Primarily Non-English speaking	19 (2.1)	10 (1.1)	5 (2.6)	1 (0.5)	—	—
Not listed	508 (56.8)	13 (1.5)	88 (46.3)	3 (1.4)	2 (4.3)	2 (2.7)
Live in SD	574 (64.2)	541 (61.3)	111 (58.4)	127 (61.1)	26 (55.3)	46 (62.2)
Live in MN	81 (9.1)	40 (4.5)	24 (12.6)	8 (3.8)	7 (14.9)	3 (4.1)
Live in IA	80 (8.9)	86 (9.8)	24 (12.6)	34 (16.3)	5 (10.6)	11 (14.9)
Live in NE	44 (4.9)	68 (7.7)	10 (5.3)	23 (11.1)	3 (6.4)	8 (10.8)
Live in ND	11 (1.2)	22 (2.5)	1 (0.5)	6 (2.9)	1 (2.1)	4 (5.4)
Live in WY	4 (0.4)	9 (1.0)	—	1 (0.5)	—	—
Non-neighboring state	100 (11.2)	116 (13.2)	20 (10.5)	9 (4.3)	5 (10.6)	2 (2.7)
Live in rural area	—	—	—	—	18	37
Close friends	238 (26.6)	250 (28.3)	39 (20.5)	48 (23.1)	10 (21.3)	15 (20.3)
Relatives	400 (44.7)	297 (33.7)	93 (48.9)	90 (43.3)	22 (46.8)	30 (40.5)
Spouse/significant other	79 (8.8)	45 (5.1)	26 (13.7)	20 (9.6)	8 (17.0)	11 (14.9)
Acquaintance	59 (6.6)	142 (16.1)	11 (5.8)	21 (10.1)	3 (6.4)	6 (8.1)
Stepfamily/in-law	54 (6.0)	24 (2.7)	11 (5.8)	4 (1.9)	3 (6.4)	2 (2.7)
Nondirected donations	25 (2.8)	119 (13.5)	9 (4.7)	23 (11.1)	1 (2.1)	9 (12.2)
Not listed	40 (4.5)	5 (0.6)	1 (0.5)	2 (1.0)	—	1 (1.4)
High school diploma/GED	24 (2.7)	312 (35.4)	12 (6.3)	61 (29.3)	5 (10.6)	16 (21.6)
Associate's degree	11 (1.2)	225 (25.5)	5 (2.6)	49 (23.6)	3 (6.4)	22 (29.7)
Bachelor's degree	67 (7.5)	227 (25.7)	27 (14.2)	64 (30.8)	14 (29.8)	21 (28.4)
Graduate degree	26 (2.9)	84 (9.5)	15 (7.9)	29 (13.9)	7 (14.9)	14 (18.9)
Other (not listed + some colleges)	766 (85.7)	34 (3.9)	131 (68.9)	6 (2.9)	18 (38.3)	1 (1.4)
Daily alcohol use	33 (3.7)	124 (14.1)	6 (3.2)	33 (15.9)	2 (4.3)	11 (14.9)
Occasional alcohol use	387 (43.3)	414 (46.9)	139 (73.2)	151 (72.6)	34 (72.3)	53 (71.6)
Previous alcohol user	61 (6.8)	31 (3.5)	13 (6.8)	5 (2.4)	2 (4.3)	2 (2.7)
Does not drink	106 (11.9)	85 (9.6)	30 (15.8)	20 (9.6)	9 (19.1)	8 (10.8)
Not listed	307 (34.3)	228 (25.9)	2 (1.1)	—	—	—
Smoker	163 (18.2)	155 (17.6)	20 (10.5)	24 (11.5)	4 (8.5)	5 (6.8)
Previous smoker	150 (16.8)	280 (31.7)	36 (18.9)	60 (28.8)	8 (17.0)	22 (29.7)
Does not smoke	432 (48.3)	439 (49.8)	132 (69.5)	124 (59.6)	35 (74.5)	47 (63.5)
Not listed	149 (16.7)	8 (0.9)	2 (1.1)	1 (0.5)	—	—
Illicit drug user	24 (2.7)	59 (6.7)	6 (3.2)	10 (4.8)	3 (6.4)	3 (4.1)
Previous user	43 (4.8)	66 (7.5)	13 (6.8)	12 (5.8)	3 (6.4)	5 (6.8)
Does not use	522 (58.4)	748 (84.8)	168 (88.4)	185 (88.9)	41 (87.2)	66 (89.2)
Not listed	305 (34.1)	9 (1.0)	3 (1.6)	1 (0.5)	—	—

Abbreviations: BMI = body mass index, GED = general educational development, IA = Iowa, MN = Minnesota, ND = North Dakota, NE = Nebraska, NKR = National Kidney Registry, SD = South Dakota, SD = standard deviation, and WY = Wyoming.

## Data Availability

The data that support the findings of this study are available from the corresponding author upon reasonable request.
